# Enhancement by Hydrogen Peroxide of Calcium Signals in Endothelial Cells Induced by 5-HT1B and 5-HT2B Receptor Agonists

**DOI:** 10.1155/2019/1701478

**Published:** 2019-02-11

**Authors:** Pavel V. Avdonin, Alexander D. Nadeev, Galina Yu. Mironova, Irina L. Zharkikh, Piotr P. Avdonin, Nikolay V. Goncharov

**Affiliations:** ^1^Koltsov Institute of Developmental Biology RAS, Moscow, Russia; ^2^Sechenov Institute of Evolutionary Physiology and Biochemistry RAS, Saint Petersburg, Russia; ^3^Institute of General Pathology and Pathophysiology RAMS, Moscow, Russia

## Abstract

Hydrogen peroxide, formed in the endothelium, acts as a factor contributing to the relaxation of blood vessels. The reason for this vasodilatory effect could be modulation by H_2_O_2_ of calcium metabolism, since mobilization of calcium ions in endothelial cells is a trigger of endothelium-dependent relaxation. The aim of this work was to investigate the influence of H_2_O_2_ on the effects of Ca^2+^-mobilizing agonists in human umbilical vein endothelial cells (HUVEC). We have found that H_2_O_2_ in concentration range 10-100 *μ*M increases the rise of [Ca^2+^]_i_ induced by 5-hydroxytryptamine (5-HT) and carbachol and does not affect the calcium signals of ATP, agonist of type 1 protease-activated receptor SFLLRN, histamine and bradykinin. Using specific agonists of 5-HT1B and 5-HT2B receptors CGS12066B and BW723C86, we have demonstrated that H_2_O_2_ potentiates the effects mediated by these types of 5-HT receptors. Potentiation of the effect of BW723C86 can be produced by the induction of endogenous oxidative stress in HUVEC. We have shown that the activation of 5-HT2B receptor by BW723C86 causes production of reactive oxygen species (ROS). Inhibitor of NADPH oxidases VAS2870 suppressed formation of ROS and partially inhibited [Ca^2+^]_i_ rise induced by BW723C86. Thus, it can be assumed that vasorelaxation induced by endogenous H_2_O_2_ in endothelial cells partially occurs due to the potentiation of the agonist-induced calcium signaling.

## 1. Introduction

In the early 1930s, the phenomenon of oxidative (respiratory) burst at phagocytosis was described [1]. This work initiated enormous number of studies of the role of ROS in biological processes and systems. High concentrations of ROS normally are characteristic function of the so-called professional phagocytes—cells of innate immunity; in other cells, high concentration of ROS is a sign of oxidative stress and a cause of cell death [2]. Low concentrations of ROS are permanently formed in almost all cells of the body and perform the functions of second messengers in redox-sensitive signaling pathways [3, 4]. The vascular endothelium plays a crucial role in maintaining homeostasis, and it is often both the main target and one of the sources of ROS [5, 6]. In physiological conditions, the synthesis of ROS by NADPH oxidases (NOX) carries out a signaling function [7]; however, the disturbance of its metabolism and associated signaling pathways is the cause of many vascular pathologies [8]. In terms of signaling, H_2_O_2_ is considered as the most stable and important kind of ROS. According to some data, the concentration of both endogenous and exogenous H_2_O_2_ can reach hundred micromoles [9–11]. In vascular endothelial cells H_2_O_2_ is produced by NOX4, and it was demonstrated that its targeted overexpression in endothelial cells results in a decrease in blood pressure and potentiation of relaxation under the action of acetylcholine and histamine [12]. It was assumed that the cause of vasodilation is hyperpolarization of the plasma membrane of smooth muscle cells induced by H_2_O_2_. However, there are some indications that H_2_O_2_ can cause the relaxation of vessels, acting directly on endothelial cells. In a number of studies, it was shown that exogenous H_2_O_2_ caused the increase of [Ca^2+^]_i_ in endothelial cells and modulated calcium signaling in response to physiological agonists [13–17]. The increase of [Ca^2+^]_i_ in endothelial cells induced by H_2_O_2_ can amplify the synthesis and release of relaxing factors. The data on the influence of H_2_O_2_ on calcium metabolism in endothelial cells are controversial. According to [16], H_2_O_2_ inhibits agonist-induced [Ca^2+^]_i_ rise in EC. However, such effects are observed at very high unphysiological concentrations of H_2_O_2_ [13]. Recently, we demonstrated that H_2_O_2_ in concentration range of 10 to 100 *μ*M stimulated mobilization of Ca^2+^ in HUVEC partially due to the activation of two-pore calcium channels [18]. Calcium ions released from endolysosomal vesicles via two-pore channels are operating as a trigger promoting Ca^2+^ mobilization from more capacious intracellular calcium depots. The aim of this study was to estimate how H_2_O_2_ influences upon the action of Ca^2+^-mobilizing agonists in HUVEC.

## 2. Materials and Methods

### 2.1. Reagents

The reagents used were as follows: BW723C86 and CGS12066B from Tocris; DCFH-DA and CalciumGreen/AM from Molecular Probes; serotonin, bradykinin, N-Acetyl-L-cysteine, VAS2870, Na_3_VO_4_, and BVT948 from Sigma-Aldrich.

### 2.2. Cell Culture

The endothelial cells were isolated from the human umbilical vein as described previously [19] with modifications [20]. The umbilical veins were washed with Hanks balanced salt solution containing antibiotics, filled with a M199 medium supplied with 0.1% collagenase (Sigma-Aldrich), and incubated at room temperature for 40 min. The cells were washed and grown in plastic vessels covered with 0.2% gelatin in M199 medium. The medium contained Earle's salts, 20% fetal bovine serum (Invitrogen, United States); 300 *μ*g/mL endothelial growth supplement from the rabbit brain obtained according to [21]; 100 *μ*g/mL heparin; and 100 *μ*g/mL gentamycin.

### 2.3. Registration of [Ca^2+^]_i_ Changes

The changes in [Ca^2+^]_i_ were registered in HUVEC of 2-4 passages with the use of the fluorescent probe CalciumGreen (Thermo Fisher Scientific, USA) and a microplate spectrofluorometer Synergy 4 (BioTech, USA). The cells grown in 96-well plates were loaded with the probe at 37°C for 1 h in M199 medium, containing 1 *μ*M CalciumGreen/AM and 100 *μ*g/mL Pluronic F127. After that, the physiological salt solution (PSS, pH 7.4) containing 145 mM NaCl, 5 mM KCl, 10 mM Hepes, 1 mM MgCl_2_, 1 mM СaCl_2_, and 10 mM glucose was added to the cells. The cells were preliminarily washed with the PSS solution to remove the M199 medium. Fluorescence was registered at 485 nm (excitation) and 530 nm (emission) at room temperature. All data are represented as ratios of two values (ΔF/Fo): the increase in the fluorescence in response to an agonist (ΔF) and the basal fluorescence of the unstimulated cells (Fo). The plots show the averages of three or more measurements.

### 2.4. Detection of ROS in HUVEC

ROS generation in HUVEC was assessed using 2′,7′-dichlorodihydrofluorescein diacetate (DCFH-DA) as a probe. The cells grown in 96-well plates were incubated with 2 *μ*M of DCFH-DA during 1 h at room temperature. The formation of ROS in the cells was determined by measuring the rate of DCFH oxidation into 2′,7′-dichlorofluorescein (DCF). DCF fluorescence was measured at excitation wavelength of 485 ± 20 nm and with emitter bandpass of 527 ± 15 nm. The rate of fluorescence rise was taken as an index of ROS generation.

### 2.5. Statistics

Data are presented as mean ± SEM of 6–12 measurements. Statistical significance was calculated using Excel and MedCalc statistical software according the Student-Newman-Keuls test.

## 3. Results

**Figure 1:**
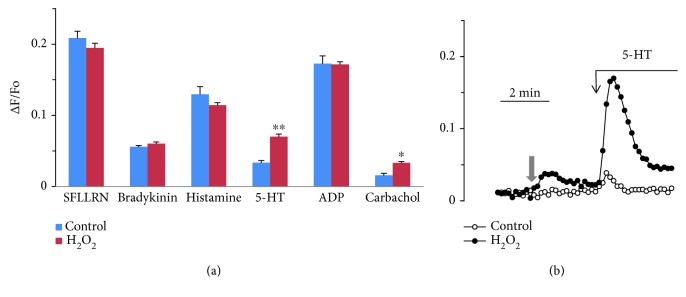
(a) Effect of the agonists on [Ca^2+^]_i_ in HUVEC in the absence and presence of H_2_O_2_. (b) Kinetics of calcium response to 5-HT in the absence and presence of H_2_O_2_. Concentration of H_2_O_2_ was 100 *μ*M. Grey arrow indicates addition of H_2_O_2_ or vehicle. Concentrations of the agonists were 5 *μ*g/mL for SFLLRN, 10^−7^ M for bradykinin, and 10^−5^ M for histamine, 5-HT, carbachol, or ADP. Each value is the mean of 6 to 9 measurements ± SEM (^∗^ *p* < 0.05; ^∗∗^ *p* < 0.01).

**Figure 2:**
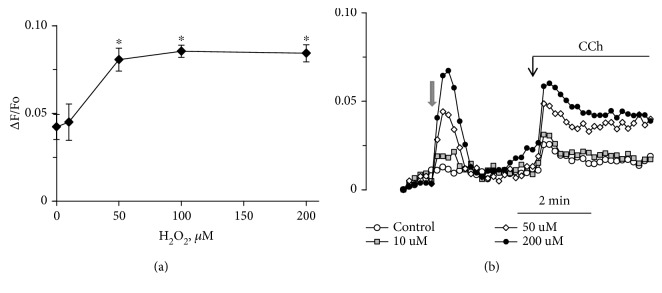
Dependence of [Ca2+]_i_ rise in HUVEC in response to 5-HT (a) or carbachol (b) on concentration of H_2_O_2_. Grey arrow indicates addition of H_2_O_2_ or vehicle in control. Concentrations of added H_2_O_2_ in (b) were 10, 50, and 200 *μ*M. The concentration of 5-HT and carbachol was 10 *μ*M. Each value in (a) is the mean of 4-6 measurements ± SEM (^∗^ *p* < 0.01).

In HUVEC, [Ca^2+^]_i_ is strongly increased in response to ADP, histamine, and the agonist of type 1 protease-activated receptor SFLLRN and to a lesser degree to bradykinin (Figure 1(a)). The responses to these agonists are not affected by H_2_O_2_. 5-hydroxytryptamine and carbachol induced substantially less elevation of [Ca^2+^]_i_ in HUVEC, and H_2_O_2_ caused a significant potentiation of their effects (Figure 1(a)). In some preparations of HUVEC, the rise of [Ca^2+^]_i_ in response to 5-HT was negligible, but in the presence of H_2_O_2_ it increased severalfold (Figure 1(b)). The reason for such strong potentiation of the [Ca^2+^]_i_ rise induced by 5-HT is not clear. We suppose that it might depend on the quality of the contacts between the cells in monolayer and the degree of synchronization of Ca^2+^ responses of single cells. The synchronization of calcium signaling of endothelial cells in monolayer has been demonstrated earlier [22]. The increase in [Ca^2+^]_i_ in response to 5-HT and carbachol occurred when the H_2_O_2_ concentration changed from 10 to 100 *μ*M (Figure 2(a)). At 200 *μ*M H_2_O_2_ did not cause additional potentiation, while its own effect on the level of [Ca^2+^]_i_ was growing (Figure 2(b)). Catalase prevents potentiation by H_2_O_2_ of 5-HT calcium signaling (Figure S1).

**Figure 3:**
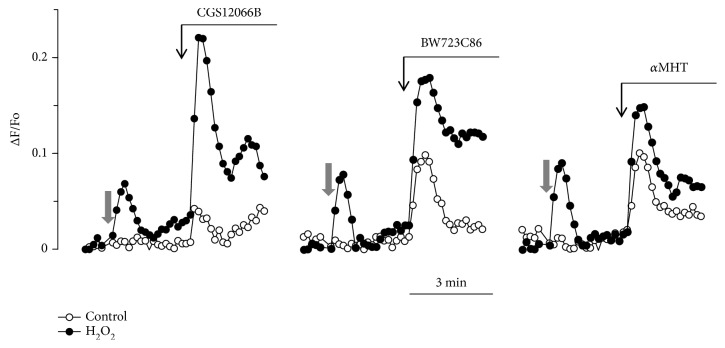
An increase in [Ca^2+^]_i_ in HUVEC in response to CGS12066B, BW723C86, and alpha-methyl-5-hydroxytryptamine (*α*MHT) upon the action of H_2_O_2_. The concentration of CGS12066B, BW723C86, and H_2_O_2_ is 50 *μ*M; *α*MHT: 10 *μ*M. Grey arrow indicates addition of H_2_O_2_ or vehicle. For each agonist, a typical response of 3-6 experiments is presented.

The vascular endothelium expresses serotonin 5-HT1B and 5-HT2B receptors. It is known that serotonin or its selective agonists activate NO synthase and cause vascular relaxation [23, 24]. In general, serotonin receptors of endothelial cells are very poorly investigated. There is no information in the literature on the participation of 5-HT1B receptors in the regulation of [Ca^2+^]_i_ in endothelial cells. Regarding 5-HT2B receptors, there is an evidence that their activation in human pulmonary artery endothelial cells causes elevation of [Ca^2+^]_i_ [25]. We investigated effects of 5-HT1B and 5-HT2B receptor agonists CGS12066B and BW723C86 in order to determine which of these receptors could be responsible for the enhancement of the Ca^2+^-mobilizing activity by H_2_O_2_ (Figure 3). The 5-HT1B receptor agonist CGS12066B caused a very weak rise of [Ca^2+^]_i_. However, its effect sharply increased in the presence of H_2_O_2_. BW723C86 at a concentration of 50 *μ*M induced the elevation of [Ca^2+^]_i_ and H_2_O_2_ further enhanced its effect near twofold. Alpha-methyl-5-hydroxytryptamine (*α*MHT) is a relatively specific agonist toward 5-HT2B receptors and its effect was also enhanced by H_2_O_2_. These results indicate that the Ca^2+^-mobilizing activity of both receptors is potentiated in the presence of H_2_O_2_.

**Figure 4:**
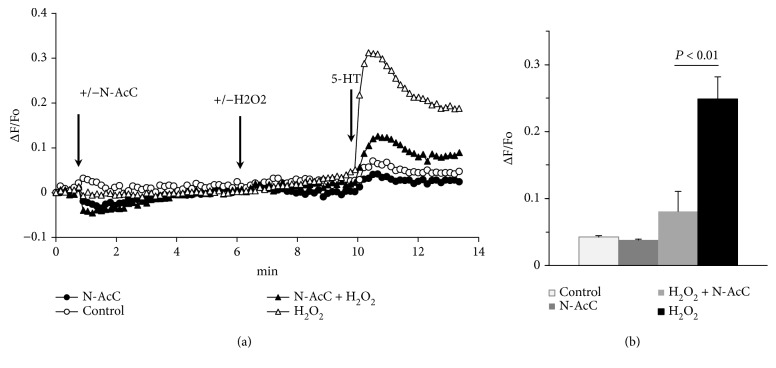
Suppression by N-acetyl cysteine (N-AcC) of H_2_O_2_-induced potentiation of [Ca^2+^]_i_ rise in response to 5-HT. Concentrations of H_2_O_2_, N-AcC, and 5-HT were 50 *μ*M, 2 mM, and 10 *μ*M, respectively.

**Figure 5:**
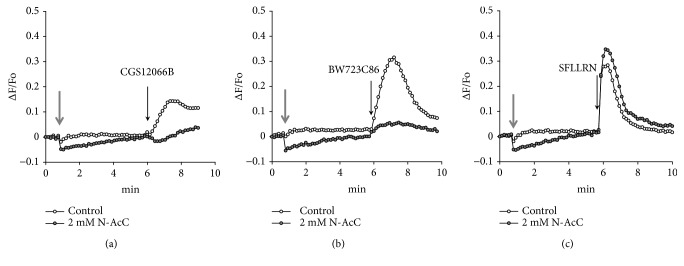
Effect of N-acetyl cysteine on [Ca2+]_i_ elevation in response to CGS12066B (50 *μ*M), BW723C86 (100 *μ*M), and SFLLRN (10 *μ*g/mL). Typical data of 3-4 experiments are presented.

**Figure 6:**
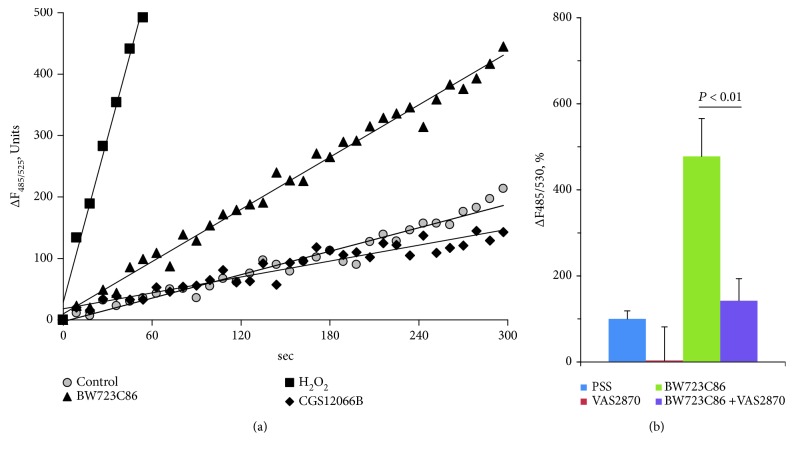
(a) Increase in the fluorescence of DCFH in HUVEC in control conditions, with the addition of exogenous H_2_O_2_ (200 *μ*M), BW723C86 (100 *μ*M), or CGS12066B (100 *μ*M). The data of one of four independent experiments are presented. (b) Inhibition of BW723C86-induced formation of ROS by inhibitor of NADPH oxidase VAS2870. The concentrations were H_2_O_2_ 200 *μ*M, BW723C86 and CGS12066B 100 *μ*M, and VAS2870 10 *μ*M.

The increase in [Ca^2+^]_i_ caused by the agonists in the presence of H_2_O_2_ does not disappear with the removal of calcium ions from the extracellular medium, which indicates mobilization of calcium ions from the intracellular depots (Figure S2). The withdrawal of extracellular calcium did not affect the effective concentration of H_2_O_2_. The full potentiation of [Ca^2+^]_i_ rise in response to CGS12066B and BW723C86 was observed at 50 *μ*M H_2_O_2_ both at normal and low level of extracellular Ca^2+^ (Figure S3) as in the case of 5-HT (Figure 2(a)). The potentiating effect of H_2_O_2_ is suppressed by the antioxidant N-acetylcysteine (Figures 4 and 5). In some HUVEC preparations, the calcium signals in response to CGS12066B and BW723C86 were quite strong. We suggested that this may be due to the formation of endogenous ROS, which might increase the calcium-mobilizing activity of 5-HT1B and 5-HT2B receptors in a manner similar to exogenous H_2_O_2_. In favor of this point of view, there is a data on the inhibition by N-acetylcysteine of [Ca^2+^]_i_ rise in response to CGS12066B and BW723C86 (Figure 6). On the other hand, there was no suppression of the effect caused by the agonist of type 1 protease-activated receptor SFLLRN. These data assume the existence of a specific mechanism through which an increase in [Ca^2+^]_i_ occurs when 5-HT1B and 5-HT2B receptors are activated in HUVEC.

In order to test the hypothesis of an increase in the functional activity of 5-HT1B and 5-HT2B receptors under the action of endogenous ROS, we investigated whether their formation in HUVEC occurred in conditions of [Ca^2+^]_i_ measurement. For registration of the ROS, the fluorescent probe DCFH-DA penetrating into the cells was used. The increase in the fluorescence due to oxidation DCFH into 2′,7′-dichlorofluorescein (DCF) reflects the accumulation of ROS in the cytoplasm. As can be seen in Figure 6(a), spontaneous formation of ROS occurs in HUVEC. In the presence of CGS12066B, the oxidation rate of the H2DCF does not change. We found that BW723C86 at a concentration of 100 *μ*M increased the formation of ROS severalfold in HUVEC (Figure 6). VAS2870, a selective inhibitor of NOX, almost completely blocked the formation of ROS induced by BW723C86 (Figure 6(b)). Thus, the data suggest the coupling of 5-HT2B receptors with NOX.

**Figure 7:**
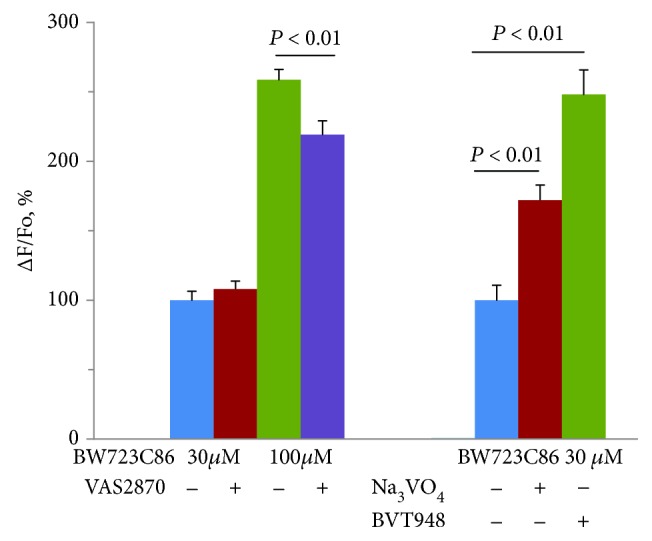
Inhibition of calcium signaling mediated by 5-HT2B receptors by VAS2870 and potentiation by Na_3_VO_4_ and BVT948. Concentrations of VAS2870, Na_3_VO_4_, and BVT948 were 10, 200, and 1 *μ*M, respectively.

The next task was to find out whether endogenous ROS generated by NOX can affect the calcium signaling in HUVEC. As shown in Figure 7(a), the NOX inhibitor VAS2870 does not change the response to 30 *μ*M BW7323C86 and decreases by 15.4 + 3.8% (*n* = 12; *p* < 0.01) the [Ca^2+^]_i_ elevation caused by BW723C86 at a concentration of 100 *μ*M. This indicates the contribution of NOX to the regulation of calcium signaling from 5-HT2B receptors. To evaluate the role of ROS endogenously formed in HUVEC in [Ca^2+^]_i_ regulation via 5-HT2B receptors, we used another approach: inducers of ROS formation were applied. Such properties are possessed by orthovanadate (Na_3_VO_4_) [26] and substance BVT948 [27]. Na_3_VO_4_ and BVT948 potentiated the rise of [Ca^2+^]_i_ in HUVEC caused by 30 *μ*M BW723C86 by 1.7 and 2.5 times (Figure 7), respectively.

## 4. Discussion

In this paper, we have demonstrated that hydrogen peroxide dramatically increases the rise of [Ca^2+^]_i_ in endothelial cells in response to serotonin. It was found that the reason for this could be an increase in the functional activity of 5-HT1B and 5-HT2B receptors. To the best of our knowledge, this is the first demonstration of Ca-mobilizing activity of 5-HT1B receptors in endothelial cells. There is only one experimental work demonstrating that 5-HT2B receptors stimulate calcium mobilization in endothelial cells, which was conducted on the cells isolated from the human pulmonary artery [25]. The reason for the lack of published data on this topic seems to be the low functional activity of 5-HT1B and 5-HT2B receptors. Earlier we showed that in smooth muscle cells of blood vessels 5-HT2B receptors are functionally inactive and their transition to the active state occurs under the influence of ROS [28]. The results presented in this paper show that the same pattern of 5-HT receptor regulation exists in HUVEC.

The increase in calcium signaling upon exposure to H_2_O_2_ is selective with respect to the action of serotonin. There is an increase in the level of [Ca^2+^]_i_ in response to carbachol, though it is extremely low even in the presence of H_2_O_2_. The difference in the effect of ROS on the functional properties of 5-HT receptors, on the one hand, and histamine, ADP, and PAR1 receptors, on the other hand, is indicative of differences in the mechanisms of signal transduction. In the paper of Ullmer et al. [25], data were presented according to which 5-HT2B receptors induce the activation of ryanodine-sensitive channels in human pulmonary endothelial cells. We have shown that the main source of increasing [Ca^2+^]_i_ in HUVEC in response to the activation of 5-HT1B and 5-HT2B receptors could certainly be intracellular stores, but their nature has not yet been established.

In many cases, H_2_O_2_ initially causes a transient rise of [Ca^2+^]_i_, but its potentiating effect can be manifested without an apparent increase in the level of [Ca^2+^]_i_ in HUVEC. In a recent paper, we showed that H_2_O_2_ activated two-pore calcium channels in HUVEC [18]. It was assumed that calcium ions released from endolysosomal vesicles through the two-pore channels serve as a trigger opening of the endo/sarcoplasmic reticulum channels. Perhaps this mechanism is implemented in this case, too.

Presently, it is a well-known fact that H_2_O_2_ at low nontoxic concentrations can serve as a second messenger. The enhancement of [Ca^2+^]_i_ is one of the terminal links in serotonergic signaling. The primary targets of H_2_O_2_ are known to be SH groups of PTEN and tyrosine phosphatases [29]. Moreover, tyrosine phosphatases are the target for different inducers of ROS formation, such as Na_3_VO_4_ and BVT946 [27, 30]. These substances have the same effect as H_2_O_2_, so there is a reason to think that the tyrosine phosphorylation system is involved in the serotonergic regulation of [Ca^2+^]_i_ in HUVEC. The involvement of tyrosine protein phosphatases and protein kinases of the Src family in calcium signaling of 5-HT2B receptors was shown in vascular smooth muscle cells [28].

In this work, we have shown that the activation of 5-HT2B receptor by BW723C86 induces ROS accumulation in the cytoplasm of HUVEC. Earlier, we showed the same effect of BW723C86 in rat aorta smooth muscle cells [28]. The formation of ROS in both cell types is suppressed specifically by the NADPH oxidase inhibitor VAS2870. This suggests the coupling of 5-HT2B receptors with this enzyme. The question about the type of NOX interacting with the 5-HT2B receptors requires further investigation.

Based on the data obtained, it might be suggested that the excessive production of H_2_O_2_ by leukocytes as well as the formation of endogenous ROS in endothelial cells could influence the serotoninergic regulation of vascular tone and secretion from endothelial cells. Serotonin stimulates von Willebrand factor secretion via 5-HT1B receptors and the mechanism of this process is not completely clear [31]. Oxidative stress and simultaneous release of 5-HT from platelets could be a pathogenic factor of microvascular thrombosis. The stimulation of 5-HT2B receptors in endothelial progenitors by nordexfenfluramine and some other pharmacological agents causes valvular heart disease [32]. The potentiation of calcium response via 5-HT2B receptors in progenitors by H_2_O_2_ can probably accelerate its development. On the other hand, the moderate formation of H_2_O_2_ in endothelial cells can cause a physiologically relevant increase of [Ca^2+^]_i_ and promote vascular relaxation. Future work will be required to uncover whether H_2_O_2_ potentiates vasorelaxation via 5-HT receptors.

## Data Availability

The data used to support the findings of this study are available from the corresponding author upon request.

## Supplementary Materials

Supplementary Materials Figure S1: inhibition by catalase of H_2_O_2_-induced [Ca^2+^]_i_ elevation and its effect on 5-HT-induced calcium signaling in HUVECs. (A) Kinetics of [Ca^2+^]_i_ changes. (B) Decrease in 5-HT-induced calcium signal. Catalase at concentration 150 units/mL or buffer was added 5 min before H_2_O_2_. Concentrations of H_2_O_2_ and 5-HT were 100 and 10 *μ*M, respectively. ^∗^ *p* < 0.01 compared to control without H_2_O_2_, *n* = 4. Figure S2: the increase in [Ca^2+^]_i_ in HUVEC incubated with 200 *μ*M H_2_O_2_ in response to CGS12066A (50 *μ*M) and BW723C86 (100 *μ*M) in a medium with calcium ions (1.25 mM CaCl_2_) and in a medium without calcium in the presence of 100 *μ*M EGTA. The average of 3 parallel measurements ± SEM is presented. Figure S3: the influence of different concentrations of H_2_O_2_ on [Ca^2+^]_i_ elevation in HUVECs induced by 50 *μ*M CGS12066B (A) and 30 *μ*M BW723C86 (B). The magnitude of the response in the presence of calcium ions and in the absence of H_2_O_2_ is taken as 100%. Each value is a mean of 6 measurements. The increments of ΔF/Fo in the presence of H_2_O_2_ significantly differ from control values (*p* < 0.01).
