# Supplementary material for: Enhancement by Hydrogen Peroxide of Calcium Signals in Endothelial Cells Induced by 5-HT1B and 5-HT2B Receptor Agonists
Source: Oxid Med Cell Longev. 2019 Feb 11;2019:1701478. doi: 10.1155/2019/1701478 (PMC6388333; doi:10.1155/2019/1701478)
Supplement: Supplementary Materials — Figure S1: inhibition by catalase of H2O2-induced [Ca2+]i elevation and its effect on 5-HT-induced calcium signaling in HUVECs. (A) Kinetics of [Ca2+]i changes. (B) Decrease in 5-HT-induced calcium signal. Catalase at concentration 150 units/mL or buffer was added 5 min before H2O2. Concentrations of H2O2 and 5-HT were 100 and 10 μM, respectively. ∗ p < 0.01 compared to control without H2O2, n = 4. Figure S2: the increase in [Ca2+]i in HUVEC incubated with 200 μM H2O2 in response to CGS12066A (50 μM) and BW723C86 (100 μM) in a medium with calcium ions (1.25 mM CaCl2) and in a medium without calcium in the presence of 100 μM EGTA. The average of 3 parallel measurements ± SEM is presented. Figure S3: the influence of different concentrations of H2O2 on [Ca2+]i elevation in HUVECs induced by 50 μM CGS12066B (A) and 30 μM BW723C86 (B). The magnitude of the response in the presence of calcium ions and in the absence of H2O2 is taken as 100%. Each value is a mean of 6 measurements. The increments of ΔF/Fo in the presence of H2O2 significantly differ from control values (p < 0.01). [file 1701478.f1.doc]

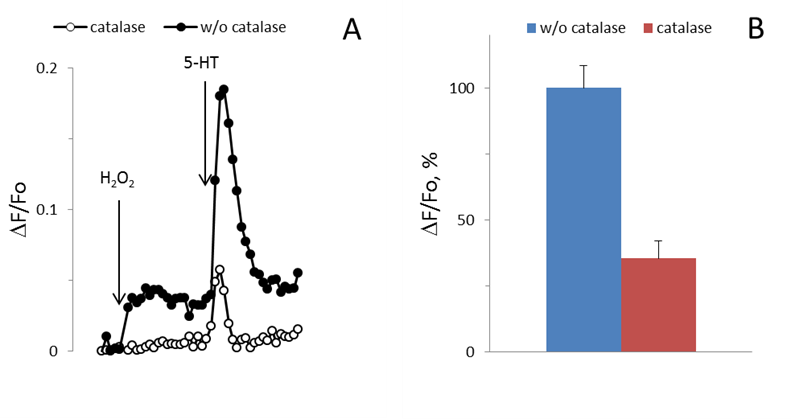


Fig S1. Inhibition by catalase of H_2_O_2_-induced [Ca^2+^]_i_ elevation and its effect on 5-HT-induced calcium signaling in HUVECs. (A) Kinetics of [Ca^2+^]_i_ changes. (B) Decrease in 5-HT-induced calcium signal. Catalase at concentration 150 units/ml or buffer were added 5 min before H_2_O_2_. Concentrations of H_2_O_2_ and 5-HT were 100 and 10 μM. *p<0.01 compared to control without H_2_O_2_, n=4.


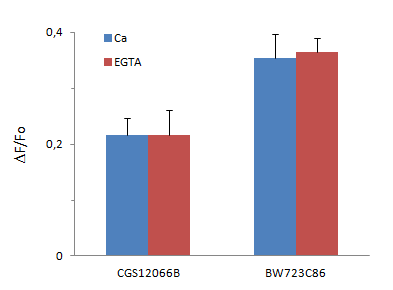


Fig.S2. The increase in [Ca^2+^]_i_ in HUVEC incubated with 200 μM H_2_O_2_ in response to CGS12066A (50 μM) and BW723C86 (100 μM) in a medium with calcium ions (1.25 mM CaCl_2_) and in a medium without calcium in the presence of 100 μM EGTA. The average of 3 parallel measurements + SEM are presented.


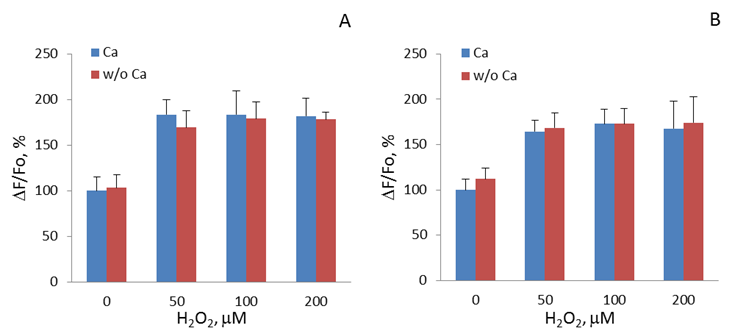


Fig.S3. The influence of different concentrations of H_2_O_2_ on [Ca^2+^]_i_ elevation in HUVECs induced by 50 μM CGS12066B (A) and 30 μM BW723C86 (B). The magnitude of the response in the presence of calcium ions and in the absence of H2O2 is taken as 100%. Each value is a mean of 6 measurements. The increments of ΔF/Fo in the presence of H_2_O_2_ significantly differ from control values (p<0.01).
